# Left atrial appendage function and structure predictors of recurrent atrial fibrillation after catheter ablation: A meta-analysis of observational studies

**DOI:** 10.3389/fcvm.2022.1009494

**Published:** 2022-10-20

**Authors:** Shaojie Han, Ming Liu, Ruikun Jia, Zhifu Cen, Ran Guo, Guobin Liu, Kaijun Cui

**Affiliations:** ^1^Department of Cardiology, West China Hospital, Sichuan University, Chengdu, China; ^2^Interventional Operating Room, Department of Cardiology, West China Hospital, Sichuan University, Chengdu, China; ^3^Department of Cardiology, The First People’s Hospital of Jintang County, Chengdu, China

**Keywords:** atrial fibrillation, atrial fibrillation recurrence, catheter ablation, meta-analysis, left atrial appendage structure, left atrial appendage function

## Abstract

**Background:**

The results of studies evaluating the left atrial appendage (LAA) function and structure as predictors of atrial fibrillation (AF) recurrence after catheter ablation (CA) are contradictory. Therefore, we performed a meta-analysis to assess whether the LAA function and structure can predict the recurrence of AF after CA.

**Methods:**

The PubMed, EMBASE, Web of Science, and Cochrane library databases were used to conduct a comprehensive literature search. Finally, 37 studies encompassing 11 LAA parameters were included in this meta-analysis.

**Results:**

Compared with those in the non-recurrence group, the recurrence group had increased LAA volume (SMD 0.53, 95% CI [0.36, 0.71] *p* < 0.00001), LAA volume index, LAA orifice area, and LAA orifice short/long axis and decreased LAA emptying flow velocity (SMD -0.54, 95% CI [-0.68, -0.40], *P* < 0.00001), LAA filling flow velocity, and LAA ejection fraction, while there was no significant difference in LAA morphology or LAA depth.

**Conclusion:**

Large LAA structure of pre-ablation (LAA volume, orifice area, orifice long/short axis, and volume index) and decreased LAA function of pre-ablation (LAA emptying flow velocity, filling flow velocity, ejection fraction, and LASEC) increase the odds of AF recurrence after CA.

**Systematic review registration:**

[https://www.crd.york.ac.uk/prospero/], identifier [CRD42022324533].

## Introduction

The most prevalent chronic cardiac arrhythmia, atrial fibrillation (AF), causes increased morbidity and death (1, 2). AF, especially persistent AF, still has a high recurrence rate, despite the use of catheter ablation (CA) as a medical therapy for it (3). Many individuals with pulmonary vein reconnection do not experience AF recurrence after pulmonary vein isolation, which implies that there are complicated underlying mechanisms beyond pulmonary vein triggers that incite AF recurrence (4, 5). Therefore, assessing the patients’ risk of AF recurrence is critical for increasing the benefits of CA and preventing the complications of multiple ablations. The presence of left atrial dilatation and impaired function has been linked to a high AF recurrence rate (6–8). However, the left atrial appendage (LAA), which plays an important role as an AF trigger, is poorly understood (9, 10). Di Biase et al. found that the LAA is an important site of triggers in 27% of 987 patients with repeated ablations (11). A meta-analysis has shown that LAA electrical isolation can achieve a higher rate of improvement in freedom from AF recurrence compared to standard ablation alone in patients with non-paroxysmal AF (12). LAA flow velocity has been used as surrogates of left atri reservoir and contractile function (13). In addition, the LAA also plays an important role in predicting cardioembolic stroke (14, 15).

However, there is disagreement on the LAA structure and function in predicting AF recurrence after CA (16–18). Therefore, this meta-analysis was conducted to determine whether LAA structure and function can predict the recurrence of AF after CA in daily clinical practice.

## Methods

### Search strategy and selection criteria

We systematically searched the PubMed, Cochrane Library, EMBASE, and Web of Science databases without language restriction until August 25, 2022. Simultaneously, a manual search of related references was conducted, and unpublished documents are sought on clinicaltrials.gov. The search terms were “atrial fibrillation,” “left atrial appendage,” “catheter ablation,” “left atrial appendage electrical isolation” and “recurrence.” Search details can be seen in the Supplementary material. The criteria for inclusion were as follows: (i) surgical ablation of AF compared to CA, the study subjects were very different; to minimize variability among study patients, we selected only patients with CA; (ii) AF recurrence after CA was measured as an outcome; (iii) 12-lead ECG or Holter ECG confirmation of AF, atrial flutter, or atrial tachycardia; and (iv) the recurrence and non-recurrence groups’ means and standard deviations of LAA parameters were provided or could be converted from the medians and ranges (19). The criteria for exclusion were as follows: (i) animal research; (ii) conference abstracts, review articles, case reports, and letters/reports, (iii) studies that included LAA parameters that had been explored in fewer than three studies. (iv) follow-up less than 3 months. The review protocol has been registered in the PROSPERO (registration number: CRD42022324533).

### Data extraction and quality appraisal

The following information was gathered from eligible studies: (i) name of the first author, publication year, and design of the research; (ii) detection strategies for AF recurrence, ablation details, and blanking period; (iii) mean follow-up time and baseline characteristics; and (iv) baseline characteristics of LAA. Two reviewers independently assessed the quality of each study by using the Newcastle-Ottawa Scale (Supplementary Table 1). Disagreements between the two reviewers were worked out through dialog and consultation of a third reviewer if necessary.

## Statistical analysis

Categorical variables are reported as a pooled risk ratio (RR). Continuous variables are expressed via standardized mean difference (SMD). For all outcomes, overall estimate with the 95% confidence interval (CI) was calculated. Cochran’s Q test and I^2^ statistics were used to assess heterogeneity. I^2^ statistics >25%, 50–75%, and >75% indicated low, moderate, or high heterogeneity, respectively. The random-effects model was used when the heterogeneity was obvious; otherwise, the fixed-effects model was used. A sensitivity analysis or subgroup analysis was performed when necessary. Subgroup analysis was used to investigate the cause of heterogeneity. R programming language (version 4.1.2, R Foundation) was used to assess publication bias by using funnel plots and Egger’s test. Review Manager Version 5.3 software (The Nordic Cochrane Centre) was used to conduct overall effect analysis and subgroup analysis.

## Results

**FIGURE 1:**
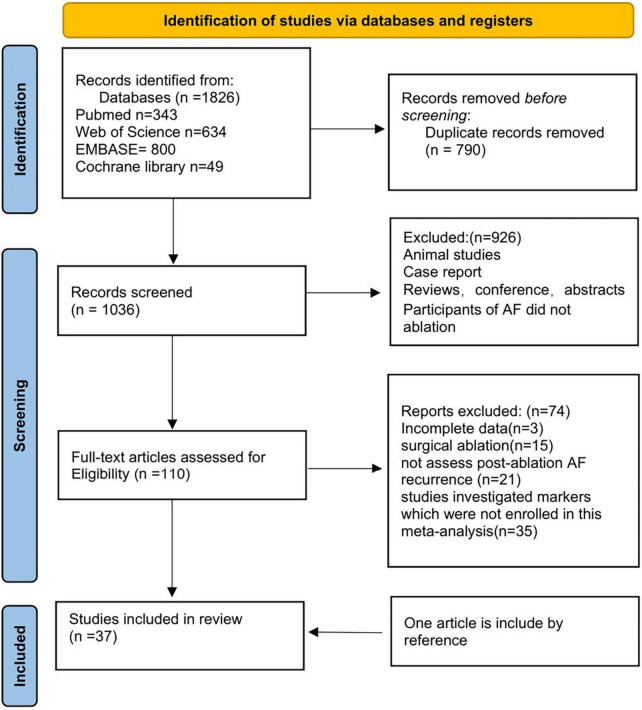
Summary of electronic search and included/excluded studies.

We retrieved 343 articles from PubMed, 49 articles from the Cochrane Library, 800 articles from EMBASE, and 643 articles from the Web of Science. 926 duplicate articles were removed from the list. Furthermore, 863 studies were excluded after reading the titles and abstracts. For the second round of selection, the entire texts of the remaining 110 studies were read: 3 articles were excluded due to incomplete data; 15 articles were excluded due to surgical ablation; 21 articles didn’t assess post-ablation AF recurrence; 35 studies didn’t investigate markers which we need in this meta-analysis. One article included was obtained from the references. Figure 1 shows a flow chart of the article screening process.

Finally, 37 observational studies were included after the application of the inclusion and exclusion criteria. The following 11 LAA parameters were covered: LAA emptying flow velocity, LAA volume, LAA filling flow velocity, LAA depth, LAA orifice long/short axis, LAA orifice area, LAA morphology, LAA volume index, LAA ejection fraction (LAAEF), and left atrial spontaneous echo contrast (LASEC). The detailed characteristics of our included patients are depicted in Supplementary Tables 2, 3.

### Left atrial appendage morphology and atrial fibrillation recurrence post-radiofrequency catheter ablation

**FIGURE 2:**
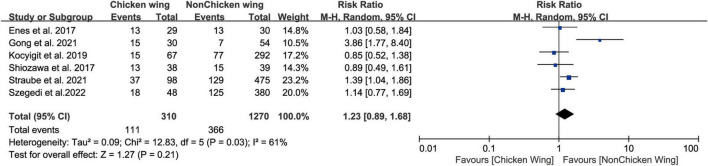
Forest plot showing the no difference in LAA morphology (chicken vs. non-chicken) between patients with and without AF recurrence after catheter ablation.

Six studies (17, 20–24) divide LAA morphology into chicken wing (CW) and no chicken wing (NCW), we did not find a statistically significant relationship between pre-ablation LAA morphology (CW vs. NCW) and post-ablation AF recurrence (Figure 2, RR 1.23, 95% CI [0.89, 1.68] *P* = 0.21). The tests for heterogeneity showed moderate heterogeneity (*I*^2^ = 61). Five studies divided the LAA into chicken wing, cauliflower, cactus, and windsock. Based on the above classification results, we found that the risk of recurrence did not differ between CW patients and windsock (Supplementary Figure 1A, RR 1.17, 95% CI[0.79, 1.72] *P* = 0.44), cactus (Supplementary Figure 1B, RR 1.04, 95% CI[0.76, 1.41] *P* = 0.81), or cauliflower (Supplementary Figure 1C, RR 1.10, 95% CI [0.85, 1.41] *P* = 0.48), patients.

### Left atrial appendage volume and atrial fibrillation recurrence catheter ablation

**FIGURE 3:**
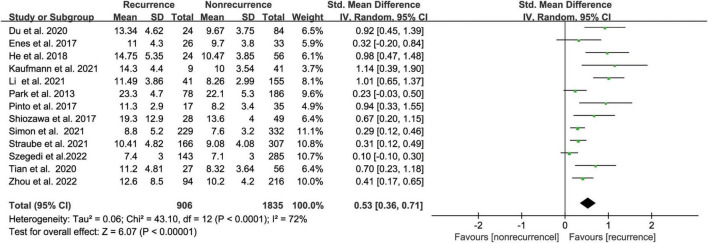
Forest plot showing the difference in LAA volume between patients with and without AF recurrence after catheter ablation.

The meta-analysis comprised thirteen (17, 18, 20, 22, 24–32) studies that evaluated the risk of AF recurrence following CA based on LAA volume in 2741 people. LAA volume was assessed using computed tomography (CT) in most studies while one study used transesophageal echocardiography (TEE). The AF recurrence group had an increased LAA volume compared with the non-recurrence group, according to our meta-analysis. (SMD 0.53, 95% CI [0.36, 0.71] *p* < 0.00001, Figure 3). But the heterogeneity was significant with I^2^ = 72% (*P* < 0.00001). After subgroup analysis by follow-up time, AF type, region, and sample size (Supplementary Figure 2); we found low heterogeneity after excluding paroxysmal AF. We performed a sensitivity analysis to see determine how each study affected the results by removing one trial at a time. However, we found no source of heterogeneity.

### Left atrial appendage emptying flow velocity and atrial fibrillation recurrence after catheter ablation

**FIGURE 4:**
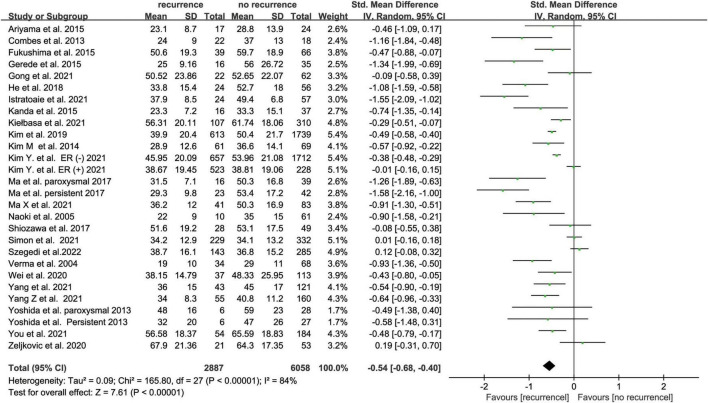
Forest plot showing the difference in LAA emptying flow velocity between patients with and without AF recurrence after catheter ablation.

Twenty-five studies (16, 17, 20, 23, 27, 33–52) with 8945 subjects about pre-ablation LAA emptying flow velocity and AF recurrence after ablation recurrence were included. LAA emptying flow velocity was assessed using TEE in most studies while one study used intracardiac echocardiogram (ICE). The recurrence group showed a lower LAA emptying flow velocity than the non-recurrence group, according to our findings (SMD -0.54 95% CI [-0.68, -0.40], *P* < 0.00001; Figure 4). The heterogeneity test revealed that I^2^ is 84%; we performed the subgroup analysis by size of the sample, follow-up time, AF type, and study region (Supplementary Figure 3), but the heterogeneity didn’t decrease. We performed a sensitivity analysis to see determine how each study affected the results by removing one trial at a time. However, we found no studies that led to heterogeneity.

### Left atrial appendage orifice area or orifice long/short axis and atrial fibrillation recurrence after catheter ablation

**FIGURE 5:**
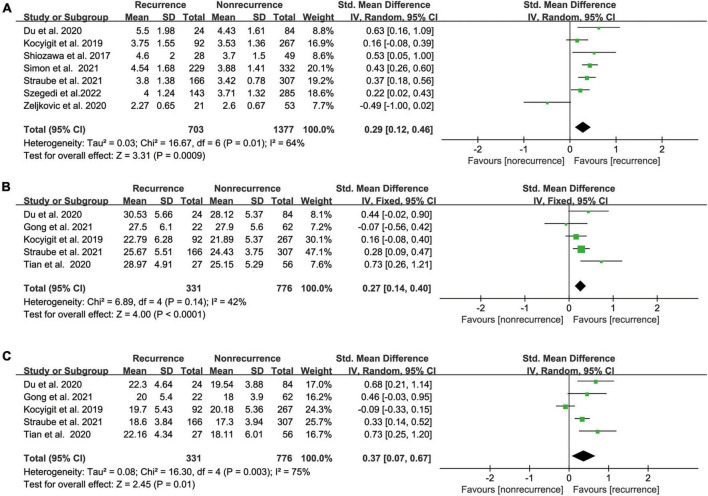
**(A)** Forest plot showing the difference in LAA orifice area between patients with and without AF recurrence after catheter ablation. **(B)** Forest plot showing the difference in LAA orifice long axis between patients with and without AF recurrence after catheter ablation. **(C)** Forest plot showing the difference in LAA orifice short axis between patients with and without AF recurrence after catheter ablation.

Our meta-analysis demonstrated a significant link between pre-ablation LAA orifice area and post-ablation AF recurrence based on the results of seven relevant studies (17, 18, 20–22, 30, 51) that included 2080 participants (SMD 0.29 95% CI [0.12, 0.46] *P* = 0.01; Figure 5A). The heterogeneity was significant (*I*^2^ = 64%). Our outcome was unaffected by sensitivity analysis, and heterogeneity was low after excluding Zeljkovic et al., who measured LAA orifice area by TEE, while other studies used CT to measure LAA orifice area.

Based on the findings of the five relevant studies (18, 21–23, 28), we found that the recurrence group had a longer LAA orifice long/short axis than the non-recurrence group, with pooled SMD of 0.27 and 0.37 (95% CI [0.14, 0.40] *P* < 0.0001 Figure 5B; 95% CI [0.07, 0.67] *p* = 0.01 Figure 5C), respectively. The heterogeneity test showed I^2^ values of 42% and 75%, respectively.

### Other left atrial appendage parameters and atrial fibrillation recurrence after catheter ablation

As is shown in Table 1, we also involved the other five parameters (Supplementary Figures 5A–E). Brief descriptions were as follows: pre-ablation LASEC, LAA ejection fraction, decreased LAA filling flow velocity, and increased LAA volume index were associated with AF after catheter ablation while LAA depth was not.

**TABLE 1:** Analysis of the association of left atrial appendage (LAA) parameters with the post-ablation atrial fibrillation (AF) recurrence.

LAA parameters	No. of studies	Participants	P value	Effect estimate (95% CI)	I^2^
LAA morphology, (CW vs. NCW)	6	1580	0.21	1.23 (0.89, 1.68)	61
LAA morphology, (CW vs. Windsock)	5	890	0.44	1.17 (0.79, 1.72)	65
LAA morphology, (CW vs. Cactus)	5	398	0.81	1.04 (0.76, 1.41)	0
LAA morphology, (CW vs. Cauliflower)	5	685	0.48	1.10 (0.85, 1.41)	0
LA/LAA spontaneous echo contrast	5	5622	0.0002	1.95 (1.38, 2.75)	90
LAA emptying flow velocity	25	8932	<0.00001	−0.54 (−0.68, −0.40)	84
LAA filling flow velocity	5	2687	<0.00001	−0.47 (−0.56, −0.39)	0
LAA ejection fraction	5	519	0.0001	−0.94 (−1.42, −0.46)	80
LAA volume	13	2741	<0.00001	0.53 (0.36, 0.71)	72
LAA volume index	3	1101	0.02	0.47 (0.09, 0.85)	57
LAA orifice area	7	2080	0.0009	0.29 (0.12, 0.46)	64
LAA orifice long axis	5	1107	<0.0001	0.27 (0.14, 0.40)	42
LAA orifice short axis	5	1107	0.01	0.37 (0.07, 0.67)	75
LAA depth	5	1375	0.09	0.20 (−0.03, 0.42)	70

LAA, Left atrial appendage; CW, chicken wing; NCW, no chicken wing.

### Publication bias analysis

We performed publication bias analysis when >10 studies were included. The funnel plots of LAA emptying flow velocity and volume were both asymmetrical with *P* < 0.05 for Egger’s test, which suggested that publication bias was evident. Therefore, we evaluated our results by the trim-and-fill method. After filling the studies, the adjusted results were still statistically significant for both the LAA emptying flow velocity and volume. The analysis results are shown in Supplementary Figures 6, 7.

## Discussion

This meta-analysis of observational studies assessed whether LAA structure and function could predict the recurrence of AF after CA. The main conclusions were as follows:

(i) LAA structure of pre-ablation (LAA volume, orifice area, orifice long/short axis, and volume index) was larger in the AF recurrence group compared than in the no recurrence group after CA.
(ii) LAA function of pre-ablation (LAA emptying flow velocity, filling flow velocity, ejection fraction, and LASEC) were reduced in patients with AF recurrence patients after CA compared with those without recurrence.
(iii) We found no statistically significant association between pre-ablation LAA morphology (CW vs. NCW, CW vs. cactus, CW vs. cauliflower, CW vs. windsock) and LAA depth.

Notably, this is the first meta-analysis to report the role of LAA function and structure in predicting AF recurrence following CA.

Left atrial appendage (LAA) emptying flow velocity is a commonly used indicator of evaluation LAA function. Previous studies have found that many factors can affect LAA velocity, including AF type, left atrium diameter, left atrium volume, LAA structure, and heart rhythm (52–55). The size of the left atrium is a predictor of AF recurrence after ablation in some meta-analyses (6, 7, 56). Reduced LAA flow velocity has been linked to increased left atrial size (14, 55), which could lead to a higher risk of AF recurrence due to atrial fibrosis and remodeling. Furthermore, new research suggests that left atrial dysfunction, rather than left atrial size, is a more sensitive predictor of AF recurrence (37). In addition, the LAA can play a considerable role in hemodynamics by modifying left atrial pressure-volume relationships because of its increased distensibility (57). The LAA works as a reservoir during excessive volume loading in the beating heart, acting as a barrier to keep the left atrial pressure from increasing too high (58). Therefore, LAA flow velocity was found to be a reliable indicator of contractile and reservoir function in the left atrium.

Moreover, our findings suggest a link between LAA volume and AF recurrence after ablation. Shirani et al’ s study found that AF patients have a considerably greater LAA volume than non-AF patients (59). The increased LAA volume may be similar to that of the left atrium, and both of are closely related to myocardial remodeling (22). With fibrosis and arrhythmogenicity of the LAA, the volume of the LAA can be used as a proxy for the link between left atrial volume and arrhythmogenicity (26). LAA structural alterations, in terms of both function and morphology, which precede left atrial remodeling, have been found to predict AF recurrence (60). LAA may be a far more sensitive criterion than left atrial structure or functional for predicting AF recurrence after CA (16). In addition, paroxysmal AF is typically in the early phases of left atrial remodeling. Therefore, LAA is a more sensitive marker for evaluating AF recurrence after ablation than left atrium in patients with paroxysmal AF (16, 37).

Although the changes in the LAA are closely related to the left atrium, LAA function and structure proved to be strong predictors of AF recurrence after controlling for left atrial structure and related clinical factors in our included study (29, 61). The tissue characteristics of the LAA differ from those of the left atrium and there is a large amount of pectinate muscle that can speed up atrial beats, resulting in faulty electrophysiological features between the LAA and left atrium (62, 63). These findings might indicate that remodeling of the LAA, which differ from the left atrium, plays distinct roles in AF recurrence after CA.

Fukushima et al found that morphology of the LAA is a major factor in the reduced in LAA emptying flow velocity (53). Only Gong et al. and Kocyigit et al. found that the morphology of the LAA is correlated with a higher likelihood of AF recurrence after CA among our six included studies (21, 23). Finally, we found that LAA morphology was not associated with recurrence of AF post ablation, similar to most of the studies we included. Further research may be needed to clarify the relevant mechanism.

These studies which our included suggested that LAA is an essential factor for the recurrence of AF after CA and our meta-analysis confirmed that LAA structure and function can influence AF recurrence. The LAA is viewed as an inconsequential auxiliary structure during the AF. But as the study goes on, we learn more and more in-depth things about the LAA. The LAA has a complicated architecture with large pectinate muscles and extremely varied muscle bundle orientation, which may allow slow conduction and block, as well as the development of re-entry, in contrast to the left atrium (64, 65). It is widely acknowledged that cardiovascular comorbidities like obesity and hypertension have a significant impact on left atrial remodeling and enlargement (26). Because the LAA differs from the left atrium in terms of its embryology, anatomy, and histology, it is unclear what causes it to grow larger (64, 65). This may explain why some patients may have very large LAA with small or moderately sized left atrium (26). We think that the first reason is that the LAA’s contraction and extension are more powerful than the left atrium, and it acts as a buffer to lower left atrial pressure (23). Second, the primary conduction channels for atrial electrical activity are the Marshall ligament and Bachmann beam close to the LAA. The normal electrophysiological activity of the LAA must be maintained by the efferent fibers of the sympathetic and vagus nerves. Distinct LAA architectures might result in different electrophysiological activity in the left atrium.

In our included literature, different imaging modalities were used. LAA flow velocity was measured using ultrasound, including TEE or ICE. Measurement of LAA structure, including cardiac CT and TEE. We have not found any studies comparing ICE and TEE measurements of LAA flow velocity. Anter et al. found that TEE can be replaced by ICE imaging during CA procedures (66). While TEE is the gold standard for perioperative imaging with LAA occlusion, a meta-analysis concluded that ICE is a viable and safe option (67). However, there is currently no accurate method for assessing LAA flow velocity using cardiac CT. For the measurement of the LAA structure, including cardiac CT and TEE. Study demonstrated intraobserver and interobserver reproducibility of TEE and CT measurements of LAA were also good (68). However, LAA measurements derived from TEE were smaller compared with those obtained by CT (68). Xu et al. found that the CTmax of the LAA ostium was substantially connected with the final deployed occluder size (Spearman’s rho: 0.81, *p* < 0.001), but the TEEmax of the LAA ostium was only moderately correlated with the occluder size (Spearman’s rho: 0.61, *p* < 0.001) (69). However, TEE can provide real-time three-dimensional views of the LAA, allowing it to play a key role for intraprocedural monitoring (70). When these two approaches are used to evaluate LAA size and shape, there may be additional benefits.

## Clinical implications

Catheter ablation is a well-established effective therapeutic option for AF. The success rate decreased to 55–65% for paroxysmal AF and 40–50% for persistent AF at five years after CA (71). Our study concluded that decreased LAA function (LAA emptying flow velocity, filling flow velocity, ejection fraction, and LASEC) and enlarged LAA size (LAA volume, orifice area, orifice long/short axis, and volume index) can predict AF recurrence after ablation. The LAA’s arrhythmogenic involvement in AF is becoming more widely understood and some researchers have proposed that, in addition to pulmonary vein isolation, the LAA may also be a target during CA for AF (62, 72). A meta-analysis concluded LAA electrical isolation led to a significantly higher improvement in freedom from all-atrial arrhythmia recurrence compared to standard ablation alone in individuals with non-paroxysmal AF (12). Therefore, evaluation of the LAA structure and function before ablation may help physicians make better choices for ablation strategies.

## Limitations

We must acknowledge that there are certain limitations of our review. First, our study presented publication bias, which we corrected for using the trim-and-fill method. After filling the studies, the adjusted results were still statistically significant for both the LAA emptying flow velocity and volume. We think the publication bias may be related to some negative results which weren’t reported. Second, the specific methods for the measurement of some parameters may not have been provided, which affects the final results in the studies we included. For example, the rhythm of the heart can significantly affect the flow velocity of the LAA. However, data on cardiac rhythm during TEE was not available. Third, there was moderate to high heterogeneity among studies on LAA flow velocity, LAA volume, and LAA orifice area. AF type, follow-up period, geographic location, and sample size were among the study parameters included in our subgroup analyses. Other clinical characteristics, such as comorbidities, gender, and various assessments of AF recurrence, might also contribute to heterogeneity. However, because several studies lacked relevant data, we were unable to do additional subgroup analyses. In addition, different imaging modalities may also lead to significant sources of heterogeneity. Finally, we have to admit that, like our similar type of meta-analysis, our study did not provide ROC-based cut-off values for LAA volume and emptying flow velocity.

## Conclusion

Our meta-analysis concluded that large LAA structure of pre-ablation (LAA volume, orifice area, orifice long/short axis, and volume index) and decreased LAA function of pre-ablation (LAA emptying flow velocity, filling flow velocity, ejection fraction, and LASEC) increase the odds of AF recurrence after CA. Pre-ablation assessment LAA function and structure might aid in physicians to improve treatment strategies.

## Data availability statement

The original contributions presented in this study are included in the article/Supplementary material, further inquiries can be directed to the corresponding authors.

## Author contributions

SH and ML conceived the review. SH drafted and wrote the manuscript. RJ, ZC, RG, and GL revised and edited all the version of the manuscript. KC revised the sections. All authors contributed to the manuscript revision and approved the submitted version.
