# Supplementary material for: Left atrial appendage function and structure predictors of recurrent atrial fibrillation after catheter ablation: A meta-analysis of observational studies
Source: Front Cardiovasc Med. 2022 Oct 20;9:1009494. doi: 10.3389/fcvm.2022.1009494 (PMC9632352; doi:10.3389/fcvm.2022.1009494)
Supplement: Supplementary file 1 [file Data_Sheet_1.docx]

# Supplementary material

**Supplementary Table 1. Quality assessment Newcastle-Ottawa score scale**

| **Item** | **Score** |
| --- | --- |
| **NOS score scale for cohort studies*** |  |
| **Selection** |  |
| (1) Representativeness of the exposed cohort |  |
| Truly representative of the average status in the community | 1 |
| Somewhat representative of the average status in the community | 1 |
| Selected group of users (e.g. nurses, volunteers) | 0 |
| No description of the derivation of the cohort | 0 |
| (2) Selection of the non exposed cohort |  |
| Drawn from the same community as the exposed cohort | 1 |
| Drawn from a different source | 0 |
| No description of the derivation of the non exposed cohort | 0 |
| (3) Ascertainment of exposure |  |
| Secure record (e.g. surgical records) | 1 |
| Structured interview | 1 |
| Written self report | 0 |
| No description | 0 |
| (4) Demonstration that outcome of interest was not present at start of study |  |
| Yes | 1 |
| No | 0 |
| **Comparability** |  |
| (1) Comparability of cohorts on the basis of the design or analysis |  |
| Study controls for the most important factor | 1 |
| Study controls for any additional factor | 1 |
| **Outcome** |  |
| (1) Assessment of outcome |  |
| Independent blind assessment | 1 |
| Record linkage | 1 |
| Self report | 0 |
| No description | 0 |
| (2) Was follow-up long enough for outcomes to occur? |  |
| Yes | 1 |
| No | 0 |
| (3) Adequacy of follow up of cohorts |  |
| Complete follow up - all subjects accounted for | 1 |
| Subjects lost to follow up unlikely to introduce bias | 1 |
| Follow up rate is low and no description of those lost | 0 |
| No statement | 0 |

*A study can be awarded a maximum of one score for each numbered item within the Selection and Outcome categories. A maximum of two scores can be given for Comparability.

Search strategy

#1 “atrial fibrillation” [Mesh] OR “atrial fibrillation”

#2 “left atrial appendage” OR "auricula sinistra" OR "left atrium appendage" OR "left auricle"

#3 "Catheter Ablation"[Mesh] OR ablation OR “radiofrequency ablation” OR “radiofrequency catheter ablation” OR “cryoballoon ablation” OR “cryoablation” OR " pulmonary vein isolation" OR “**left atrial appendage electrical isolation**”OR “left atrial appendage isolation”OR “electrical isolation” OR “LAA isolation”

#4 recurrence OR relapse OR predict* OR occurrence

#5 #1 AND #2 AND #3 AND #4

Supplementary Table 2 Characteristics of included studies

| **Study** | **Year** | **Region** | **Study design** | **AF type** | **No. patients** | | **Recurrence** | **No recurrence** | **Mean**  **Follow-up** | **Imaging used** | | **RFA details** | **Blanking**  **Period,**  **months** | | | **Recurrence**  **detection** | | **LAA paraments** | **NOS score** | |  |  |
| --- | --- | --- | --- | --- | --- | --- | --- | --- | --- | --- | --- | --- | --- | --- | --- | --- | --- | --- | --- | --- | --- | --- |
| Verma et al. | 2004 | USA. | Prospective | 2 | 102 | | 34 | 68 | 6 m | ICE | | 1 | 2 | | | Holter | | 1 | 6 | |  |  |
| Naoki et al. | 2005 | Japan | NR | 2 | 71 | | 10 | 61 | 28 m | TEE | | 1 | 3-6 | | | NR | | 1 | 6 | |  |  |
| Combes et al. | 2013 | France | Prospective | 2 | 40 | | 22 | 18 | 12 m | TEE | | 2 | 3 | | | Holter | | 1 | 7 | |  |  |
| Park et al. | 2013 | Korea | Retrospective | 3 | 264 | 78 | | 186 | 36 m | | CT | 2 | | 3 | Holter | | 2. 9. 11 | | | 8 | |  |
| Machino-Ohtsuka et al. | 2013 | Japan | NR | 3 | 123 | 45 | | 78 | 18 m | | TEE | 2 | | 3 | Holter | | 8 | | | 7 | |  |
| Yoshida et al. | 2013 | Japan | Retrospective | 3 | 67 | 12 | | 55 | 6 m | | TEE | 2 | | 3 | NR | | 1 | | | 6 | |  |
| Kim M et al. | 2014 | Korea | Prospective | 2 | 130 | 61 | | 69 | 24 m | | TEE | 2 | | NR | Holter | | 1. 7. 8. 9 | | | 7 | |  |
| Ariyama et al. | 2015 | Japan | Retrospective | 2 | 41 | 17 | | 24 | 12 m | | TEE | 2 | | 3 | Holter | | 1. 7 | | | 7 | |  |
| Fukushima et al. | 2015 | Japan | Prospective | 1 | 105 | 39 | | 66 | 12 m | | TEE | 2 | | 2 | Holter | | 1 | | | 8 | |  |
| Gerede et al. | 2015 | Turkey | Prospective | 1 | 51 | | 16 | 35 | 12 m | | TEE | 1 | | NR | Holter | | 1. 8 | | | 7 | | |
| Kanda et al. | 2015 | Japan | Retrospective | 2 | 53 | | 16 | 37 | 12 m | | TEE | 2 | | 3 | Holter | | 1 | | | 7 | | |
| Enes et al. | 2017 | Canada | Retrospective | 2 | 59 | | 26 | 33 | 13 m | | CT | 2 | | 3 | Holter | | 2. 10 | | | 7 | | |
| Ma et al. | 2017 | China | Prospective | 3 | 120 | | 39 | 81 | 12 m | | TEE | 1 | | NR | Holter | | 1 | | | 8 | | |
| Pinto et al. | 2017 | Portugal. | Retrospective | 3 | 52 | | 17 | 35 | 24 m | | CT | 2 | | 6 | Recorder or Holter | | 2 | | | 8 | | |
| Shiozawa et al. | 2017 | Portugal | Prospective | 3 | 77 | | 28 | 49 | 12 m | | TEE+CT | 2 | | 3 | Holter | | 1. 2. 4 | | | 8 | | |
| He et al. | 2018 | China | Prospective | 1 | 80 | | 24 | 56 | 12 m | | TEE | NR | | 3 | Holter | | 1. 2. 7. 9 | | | 6 | | |
| Kim et al. | 2019 | Korea | Retrospective | 3 | 2352 | | 613 | 1739 | 12 m | | TEE | 2 | | 3 | Holter | | 1. 7.8 | | | 8 | | |
| Kocyigit et al. | 2019 | Turkey. | Prospective | 3 | 359 | | 92 | 267 | 37^a^ m | | CT | 1 | | 3 | Holter | | 3. 4. 5. 6. 11 | | | 8 | | |
| Du et al. | 2020 | China | Retrospective | 3 | 108 | | 24 | 84 | 12 m | | CT | 2 | | 3 | Holter | | 2. 4. 5. 6. | | | 8 | | |
| Tian et al. | 2020 | China | Retrospective | 3 | 83 | | 27 | 56 | 12 m | | CT | 1 | | 3 | Holter | | 2. 5. 6. 9. 11 | | | 8 | | |
| Wei et al. | 2020 | China | Prospective | 3 | 150 | | 37 | 113 | 14 m | | TEE | 2 | | 3 | 72-hour Holter | | 1 | | | 8 | | |
| Zeljkovic et al. | 2020 | Croatia. | Prospective | 1 | 74 | | 21 | 53 | 12 m | | TEE | 2 | | 3 | Holter | | 1, 4 | | | 7 | | |
| Gong et al. | 2021 | China | Retrospective | 3 | 84 | | 22 | 62 | 618.6 d | | TEE | 2 | | 3 | Holter | | 1. 5. 6. 7 | | | 8 | | |
| Istratoaie et al. | 2021 | Romania | Prospective | 1 | 81 | | 24 | 57 | 12 m | | TEE | 1 | | 3 | Holter | | 1 | | | 7 | | |
| Kaufmann et al. | 2021 | Austria | Retrospective | 3 | 50 | | 9 | 41 | 229 d | | CT | NR | | NR | Holter | | 2. 9. 10 | | | 7 | | |
| Kiełbasa et al. | 2021 | Poland | Retrospective | 3 | 417 | | 107 | 310 | 24^a^ m | | TEE | 1 | | 3 | Holter or implanted device. | | 1 | | | 8 | | |
| Kim et al. | 2021 | Korea | Retrospective | 3 | 992 | | 362 | 630 | 36 m | | CT | 2 | | 3 | Holter | | 2 | | | 8 | | |
| Kim Y. et al. | 2021 | Korea | Retrospective | 3 | 3120 | | 1180 | 1940 | 60 m | | TEE | 2 | | 3 | Holter | | 1 | | | 8 | | |
| Li et al. | 2021 | China | NR | 1 | 196 | | 41 | 155 | 12 m | | CT | NR | | NR | Holter | | 2. 11.8 | | | 7 | | |
| Ma et al. | 2021 | China | Prospective | 3 | 124 | | 41 | 83 | 12 m | | TEE | 2 | | 3 | Holter | | 1 | | | 8 | | |
| Simon et al. | 2021 | Netherlands | Retrospective | 2 | 561 | | 229 | 332 | 12 m | | CT+TEE | 2 | | 3 | Holter | | 1, 2. 4 | | | 7 | | |
| Straube et al | 2021 | Germany. | Prospective | 3 | 473 | | 166 | 307 | 19 m | | CT+TEE | 2 | | 3 | Holter | | 2. 4. 5. 6. 11 | | | 8 | | |
| Yang et al. | 2021 | China | Retrospective | 2 | 164 | | 43 | 121 | 15^a^ m | | TEE | 2 | | 3 | Holter | | 1 | | | 7 | | |
| Yang Z et al. | 2021 | China | Prospective | 2 | 215 | | 55 | 160 | 3—6 m | | TEE | 2 | | 3 | Holter | | 1 | | | 7 | | |
| You, L. et al. | 2021 | China | Prospective | 1 | 238 | | 54 | 184 | 48 m | | TEE | 1 | | 3 | Holter | | 1 | | | 7 | | |
| Szegedi et al. | 2022 | Hungary. | Retrospective | 1 | 428 | | 143 | 285 | 12 m | | CT+TEE | 1 | | 3 | Holter | | 1. 2 4 | | | 7 | | |
| Zhou et al. | 2022 | Japan | NR | 3 | 310 | | 94 | 216 | 13.5 m | | CT | 2 | | 3 | Holter | | 2 | | | 8 | | |

AF type:1. paroxysmal AF (PAF); 2. persistent AF (Pers-AF); 3. PAF and Pers-AF/nonPAF. RFA details : Radiofrequency ablation details , 1: CPVI, circumferential pulmonary vein isolation; 2 :CPVI PLUS, includes CPVI with one or more of adjuvant ablations in cavotricuspid isthmus, mitral isthmus, left atrial roof, the basal posterior wall, superior vena cava or complex fractionate atrial electrograms; ICE: Intracardiac echocardiography; TTE, transthoracic echocardiography; CT, computed tomography(cardiac); NOS score: Newcastle-Ottawa Scale; LAA: left atrial appendage. LAA parameters:1. LAA emptying flow velocity; 2. LAA volume; 3.LAA morphology; 4. LAA orifice area; 5. LAA orifice long axis; 6.LAA orifice short axis; 7. LAA filling flow velocity; 8. Left atrial spontaneous echo contrast;9. LAA ejection fraction; 10. LAA volume index; 11.LAA depth.

a: median

Supplementary Table 3 Raw data from the individual studies for continuous variable

|  |  | Recurrence | | | Non-recurrence | | |
| --- | --- | --- | --- | --- | --- | --- | --- |
|  |  | mean | SD | total | mean | SD | total |
| Verma et al. 2004 | LAA emptying velocity cm/s | 19 | 10 | 34 | 29 | 11 | 68 |
| Naoki et al. 2005 | LAA emptying velocity cm/s | 22 | 9 | 10 | 35 | 15 | 61 |
| Combes et al. 2013 | LAA emptying velocity cm/s | 24 | 9 | 22 | 37 | 13 | 18 |
| Park et al. 2013 | LAA volume ml | 23.3 | 4.7 | 78 | 22.1 | 5.3 | 186 |
|  | LAA ejection fraction (%) | 59.1 | 13.7 | 35 | 65.5 | 13.8 | 141 |
|  | LAA depth mm | 43.6 | 8.9 | 78 | 43.1 | 9.0 | 186 |
| Yoshida et al. Paroxysmal 2013 | LAA emptying velocity cm/s | 48 | 16 | 6 | 59 | 23 | 28 |
| Yoshida et al. Persistent 2013 | LAA emptying velocity cm/s | 32 | 20 | 6 | 47 | 26 | 27 |
| Ariyama et al. 2015 | LAA emptying velocity cm/s | 23.1 | 8.7 | 17 | 28.8 | 13.9 | 24 |
|  | LAA filling velocity cm/s | 23.9 | 8.10 | 17 | 32.9 | 16 | 24 |
| Kim M et al.2014 | LAA emptying velocity cm/s | 28.9 | 12.6 | 61 | 36.6 | 14.1 | 69 |
|  | LAA filling velocity cm/s | 36.1 | 17.8 | 61 | 46.9 | 21 | 69 |
|  | LAA ejection fraction (%) | 25.2 | 11.9 | 61 | 29.5 | 11.1 | 69 |
| Fukushima et al. 2015 | LAA emptying velocity cm/s | 50.6 | 19.3 | 39 | 59.7 | 18.9 | 66 |
| Gerede et al. 2015 | LAA emptying velocity cm/s | 25 | 9.16 | 16 | 56 | 26.72 | 35 |
| Kanda et al. 2015 | LAA emptying velocity cm/s | 23.3 | 7.2 | 16 | 33.3 | 15.1 | 37 |
| Enes et al. 2017 | LAA volume ml | 11 | 4.3 | 26 | 9.7 | 3.8 | 33 |
|  | LAA volume index mL/m^2^ | 7.5 | 3.0 | 26 | 7.2 | 2.5 | 33 |
| Ma et al. 2017 Paroxysmal | LAA emptying velocity cm/s | 31.5 | 7.1 | 16 | 50.3 | 16.8 | 39 |
| Ma et al. 2017 Persistent | LAA emptying velocity cm/s | 29.3 | 9.8 | 23 | 53.4 | 17.2 | 42 |
| Pinto et al. 2017 | LAA volume ml | 11.3 | 2.9 | 17 | 8.2 | 3.4 | 35 |
| Shiozawa et al. 2017 | LAA emptying velocity cm/s | 51.6 | 19.2 | 28 | 53.1 | 17.5 | 49 |
|  | LAA volume ml | 19.3 | 12.9 | 28 | 13.6 | 4 | 49 |
|  | LAA-orifice area cm^2^ | 4.6 | 2.0 | 28 | 3.7 | 1.5 | 49 |
| He et al. 2018 | LAA emptying velocity cm/s | 33.8 | 15.4 | 24 | 52.7 | 18 | 56 |
|  | LAA filling velocity cm/s | 48.1^a^ | (24.9, 56.7)^b^ | 24 | 50.6^a^ | ( 47.4, 72.8)^b^ | 56 |
|  | LAA volume ml | 13.3^a^ | (11.3, 17.8)^b^ | 24 | 11.2^a^ | (7.5, 12.7)^b^ | 56 |
|  | LAA ejection fraction % | 40.4^a^ | (26.3, 45.3)^b^ | 24 | 49.0^b^ | (44.7, 64.9)^b^ | 56 |
| Kim et al. 2019 | LAA emptying velocity cm/s | 39.9 | 20.4 | 613 | 50.4 | 21.7 | 1739 |
|  | LAA filling velocity cm/s | 42.0 | 21.2 | 613 | 52.1 | 22.1 | 1739 |
| Kocyigit et al. 2019 | LAA orifice area, cm^2^ | 3.75 | 1.55 | 92 | 3.53 | 1.36 | 267 |
|  | LAA depth, mm | 49.45 | 10.50 | 92 | 49.54 | 10.40 | 267 |
|  | LAA orifice long axis mm | 22.79 | 6.28 | 92 | 21.89 | 5.37 | 267 |
|  | LAA orifice short axis mm | 19.70 | 5.43 | 92 | 20.18 | 5.36 | 267 |
| Du et al. 2020 | LAA volume ml | 13.34 | 4.62 | 24 | 9.67 | 3.75 | 84 |
|  | LAA-orifice area cm^2^ | 5.5 | 1.98 | 24 | 4.43 | 1.61 | 84 |
|  | LAA orifice long axis mm | 30.53 | 5.66 | 24 | 28.12 | 5.37 | 84 |
|  | LAA orifice short axis mm | 22.3 | 4.64 | 24 | 19.54 | 3.88 | 84 |
| Tian et al. 2020 | LAA volume ml | 11.2 | 4.81 | 27 | 8.32 | 3.64 | 56 |
|  | LAA ejection fraction (%) | 37.21 | 7.03 | 27 | 51.14 | 12.43 | 56 |
|  | LAA orifice long axis mm | 28.97 | 4.91 | 27 | 25.50^a^ | (21.70,28.90)^b^ | 56 |
|  | LAA orifice short axis mm | 22.16 | 4.34 | 27 | 18.11 | 6.01 | 56 |
|  | LAA depth mm | 47.01 | 10.33 | 27 | 39.10^a^ | (32.68,44.15)^b^ | 56 |
| Wei et al. 2020 | LAA emptying velocity m/s | 0.35^a^ | (0.28, 0.46)^b^ | 37 | 0.50^a^ | (0.30, 0.65)^b^ |  |
| Zeljkovic et al. 2020 | LAA emptying velocity cm/s | 67.9 | 21.36 | 21 | 64.3 | 17.35 | 53 |
|  | LAA-orifice area cm2 | 2.27 | 0.65 | 21 | 2.6 | 0.67 | 53 |
| Gong et al. 2021 | LAA emptying velocity cm/s | 50.52 | 23.86 | 22 | 52.65 | 22.07 | 62 |
|  | LAA filling velocity cm/s | 50.96 | 21.09 | 22 | 56.03 | 23.32 | 62 |
|  | LAA orifice long axis mm | 27.5 | 6.1 | 22 | 27.9 | 5.6 | 62 |
|  | LAA orifice short axis mm | 20.0 | 5.4 | 22 | 18.0 | 3.9 | 62 |
| Istratoaie et al. 2021 | LAA emptying velocity cm/s | 37.9 | 8.5 | 24 | 49.4 | 6.8 | 57 |
| Kaufmann et al. 2021 | LAA ejection fraction % | 16 | 8 | 9 | 42 | 14 | 41 |
|  | LAA volume, ml | 14.3 | 4.4 | 9 | 10 | 3.54 | 41 |
|  | LAA volume index mL/m^2^ | 7.07 | 1.87 | 9 | 4.96 | 1.87 | 41 |
| Kiełbasa et al. 2021 | LAA emptying velocity cm/s | 53^a^ | (42, 67)^b^ | 107 | 59^a^ | (49, 72)^b^ | 310 |
| Kim et al. 2021 | LAA volume index mL/m^2^ | 6.3^a^ | (4.8, 8.8)^b^ | 362 | 5.5^a^ | (4, 7.3)^b^ | 630 |
| Li et al. 2021 | LAA volume | 11.49 | 3.86 | 41 | 8.26 | 2.99 | 155 |
|  | LAA depth mm | 43.47 | 9.98 | 41 | 42.65 | 8.89 | 155 |
| Kim Y. et al. 2021 ER (+) | LAA emptying velocity cm/s | 38.67 | 19.45 | 523 | 38.81 | 19.06 | 228 |
| Kim Y. et al. 2021 ER (-) | LAA emptying velocity cm/s | 45.95 | 20.09 | 657 | 53.96 | 21.08 | 1712 |
| Ma X et al. 2021 | LAA emptying velocity cm/s | 36.2 | 12.0 | 41 | 50.3 | 16.9 | 83 |
| Simon et al. 2021 | LAA emptying velocity cm/s | 34.2 | 12.9 | 229 | 34.1 | 13.2 | 332 |
|  | LAA volume ml | 8.8 | 5.2 | 229 | 7.6 | 3.2 | 332 |
|  | LAA-orifice area mm2 | 454.4 | 167.7 | 229 | 387.6 | 140.5 | 332 |
| You et al. 2021 | LAA emptying velocity cm/s | 56.58 | 18.37 | 54 | 65.59 | 18.83 | 184 |
| Straube et al.2021 | LAA volume ml | 9.4 ^a^ | (7.1, 13.4)^b^ | 166 | 8.3^a^ | (6.3,11)^b^ | 307 |
|  | LAA-orifice area mm2 | 362.9^a^ | (280.4, 462)^b^ | 166 | 326.7^a^ | (263.9,417.8)^b^ | 307 |
|  | LAA depth, mm | 41.08 | 9.32 | 166 | 39.38 | 8.35 | 307 |
|  | LAA orifice long axis mm | 25^a^ | (21.8, 29)^b^ | 166 | 25^a^ | (22, 27)^b^ | 307 |
|  | LAA orifice short axis mm | 18^a^ | (16,21)^b^ | 166 | 16^a^ | (15,21)^b^ | 307 |
| Yang et al. 2021 | LAA emptying velocity cm/s | 36 | 15 | 43 | 45 | 17 | 121 |
| Yang Z et al. 2021 | LAA emptying velocity cm/s | 34 | 8.3 | 55 | 40.8 | 11.2 | 160 |
| You, L. et al 2021 | LAA emptying velocity cm/s | 56.58 | 18.37 | 54 | 65.59 | 18.83 | 184 |
| Szegedi et al. 2022 | LAA emptying velocity cm/s | 38.7 | 16.1 | 143 | 36.8 | 15.2 | 285 |
|  | LAA volume (ml) | 7.4 | 3 | 143 | 7.1 | 3 | 285 |
|  | LAA-orifice area mm2 | 400.2 | 123.5 | 143 | 370.8 | 132.3 | 285 |
| Zhou et al. 2022 | LAA volume ml | 12.6 | 8.5 | 94 | 10.2 | 4.2 | 216 |

a:median; b:quartile

**Supplementary Table 4 Subgroup analysis of the association of LAA volume with the post-ablation AF recurrence**

| Grouping variables | subgroup | No. of studies | SMD with 95% CI | P values for association | I^2^ (%) | P values for heterogeneity |
| --- | --- | --- | --- | --- | --- | --- |
| follow-up duration | ≤12 months | 8 | 0.67(0.38, 0.96) | 0.0001 | 81 | <0.00001 |
|  | > 12 months | 5 | 0.35(0.21, 0.49) | <0.00001 | 17 | 0.31 |
| participants type | PAF | 4 | 0.74(0.11, 1.36) | 0.02 | 88 | <0.00001 |
|  | Pers-AF | 3 | 0.30(0.14, 0.46) | 0.002 | 0 | 0.86 |
|  | PAF and Pers-AF | 7 | 0.50(0.31, 0.69) | <0.00001 | 54 | 0.04 |
| sample size | ≤100 | 8 | 0.46(0.27, 0.66) | <0.00001 | 78 | <0.0001 |
|  | ＞100 | 5 | 0.69(0.45, 0.94) | <0.00001 | 0 | 0.4 |
| region | Europe | 7 | 0.39(0.19, 0.58) | <0.0001 | 61 | 0.02 |
|  | Asia | 6 | 0.67(0.36,0.71) | <0.00001 | 73 | 0.002 |

the“PAF” subgroup included studies which only investigated the PAF patients, the “PAF and Pers-AF” subgroup included studies which investigated a mixture of PAF patients and Pers-AF/, the “Pers-AF” subgroup included studies which only investigated the Pers-AF patients,

**Supplementary Table 5 Subgroup** **analysis of the association of LAA emptying flow velocity with the post-ablation AF recurrence**

| Grouping variables | subgroup | No. of studies | SMD with 95% CI | P values for association | I^2^ (%) | P values for heterogeneity |
| --- | --- | --- | --- | --- | --- | --- |
| follow-up duration | ≤12 months | 18 | -0.66(-0.87, -0.45) | <0.00001 | 86 | <0.00001 |
|  | > 12 months | 7 | -0.34(-0.51, -0.17) | <0.0001 | 71 | 0.001 |
| participants type | PAF | 9 | -0.68(-1.11, -0.25) | 0.002 | 89 | <0.00001 |
|  | Pers-AF | 10 | -0.68(-1.00, -0.36) | <0.0001 | 82 | <0.00001 |
|  | PAF and Pers-AF | 8 | -0.38(-0.55, -0.22) | <0.00001 | 82 | <0.00001 |
| sample size | ≤100 | 12 | -0.79(-1.12, -0.46) | <0.00001 | 76 | <0.00001 |
|  | ＞100 | 13 | -0.39(-0.54,-0.24) | <0.00001 | 85 | <0.00001 |
| region | Europe | 9 | -0.50(-0.83, -0.16) | 0.004 | 89 | <0.00001 |
|  | Asia | 16 | -0.57(-0.71, -0.43) | <0.00001 | 75 | <0.00001 |

**Supplementary Table 6 Subgroup analysis of the association of LAA orifice area with the post-ablation AF recurrence**

| Grouping variables | subgroup | No. of studies | SMD with 95% CI | P values for association | I^2^ (%) | P values for heterogeneity |
| --- | --- | --- | --- | --- | --- | --- |
| follow-up duration | ≤12 months | 4 | 0.24 (-0.07, 0.55) | 0.12 | 78 | 0.003 |
|  | > 12 months | 3 | 0.31(0.13, 0.49) | 0.0009 | 28 | 0.25 |
| participants type | PAF | 2 | -0.03(-0.87, -0.81) | 0.95 | 89 | 0.002 |
|  | Pers-AF | 1 | 0.43(0.26, 0.60) | <0.00001 |  |  |
|  | PAF and Pers-AF | 4 | 0.29(0.11, 0.48) | <0.002 | 33 | 0.21 |
| sample size | ≤100 | 2 | 0.02(-0.97, 1.02) | 0.97 | 88 | 0.004 |
|  | ＞100 | 5 | 0.33(0.21, 0.45) | <0.0001 | 36 | 0.18 |

**Supplementary Figures**

Supplementary Figure 1

**
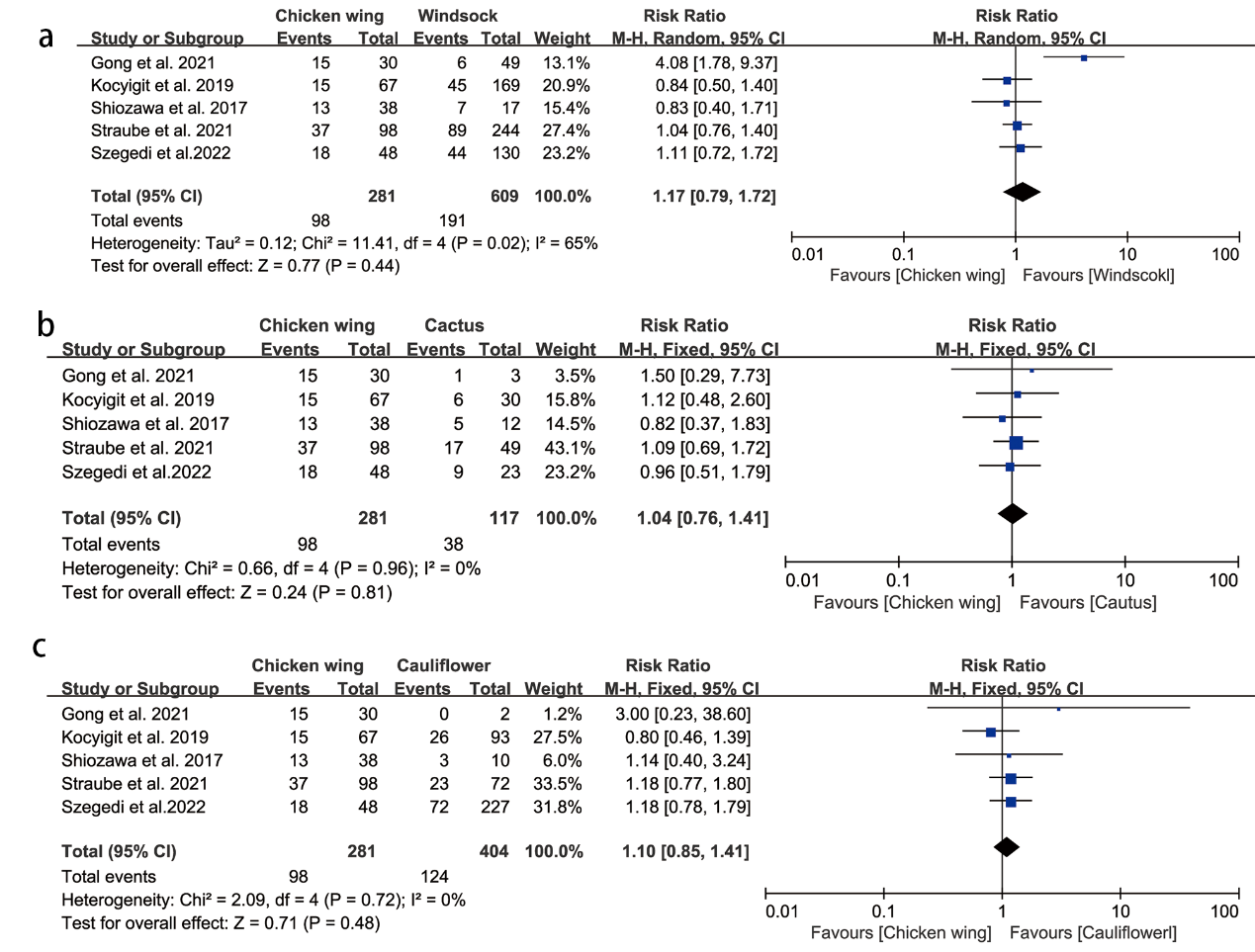
**

**Supplementary Figure 1** Analysis of the association of LAA morphology with the post-ablation AF recurrence a: Chicken wing vs. Windsock; b: Chicken wing vs. Cactus; a: Chicken wing vs. Cauliflower.

Supplementary Figure 2a


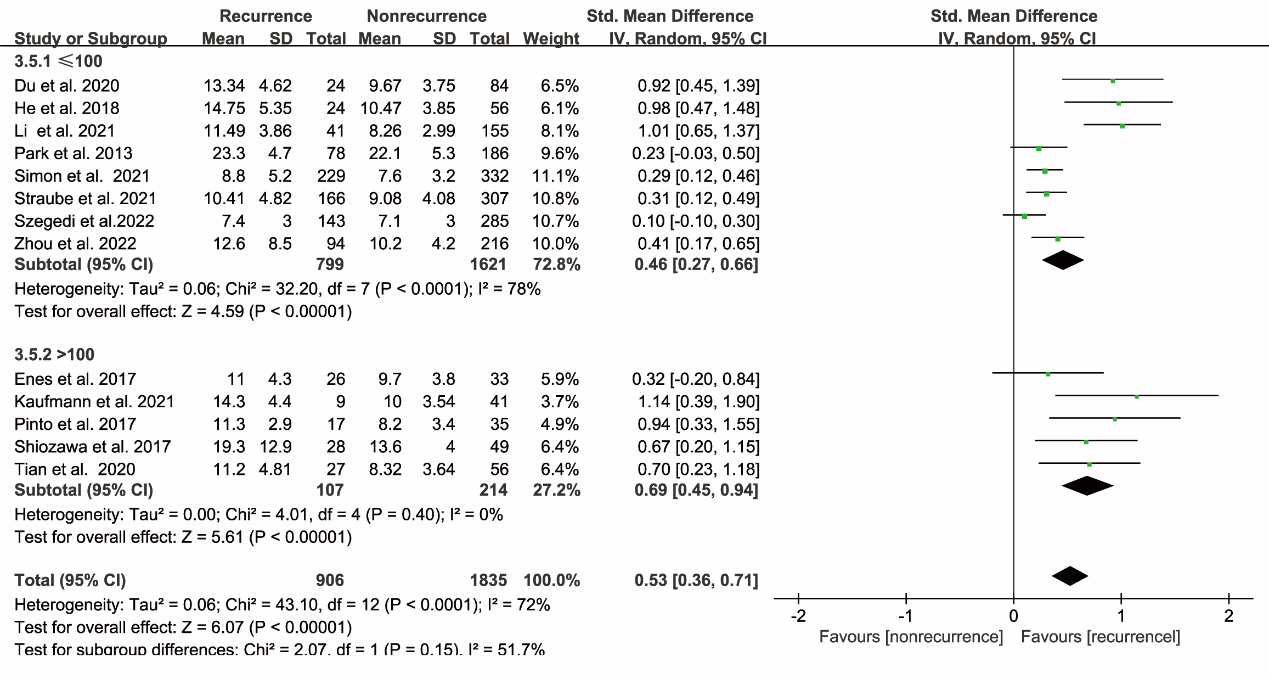


Supplementary Figure 2b


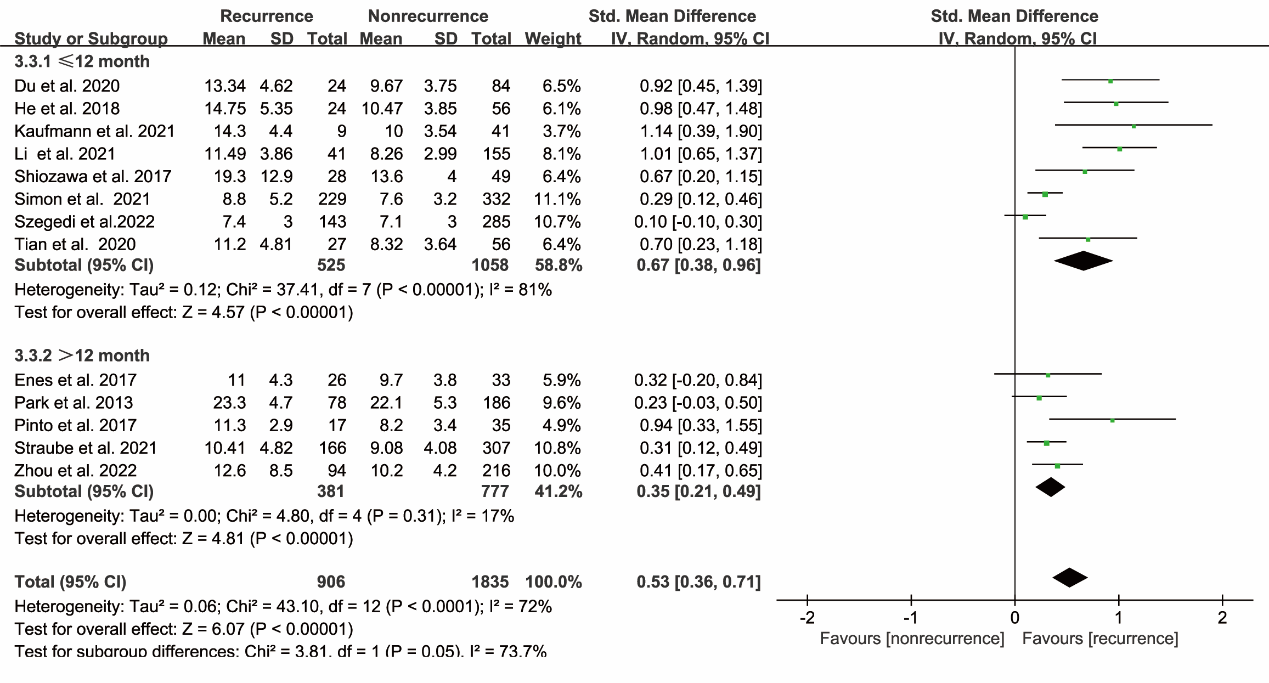


Supplementary Figure 2c


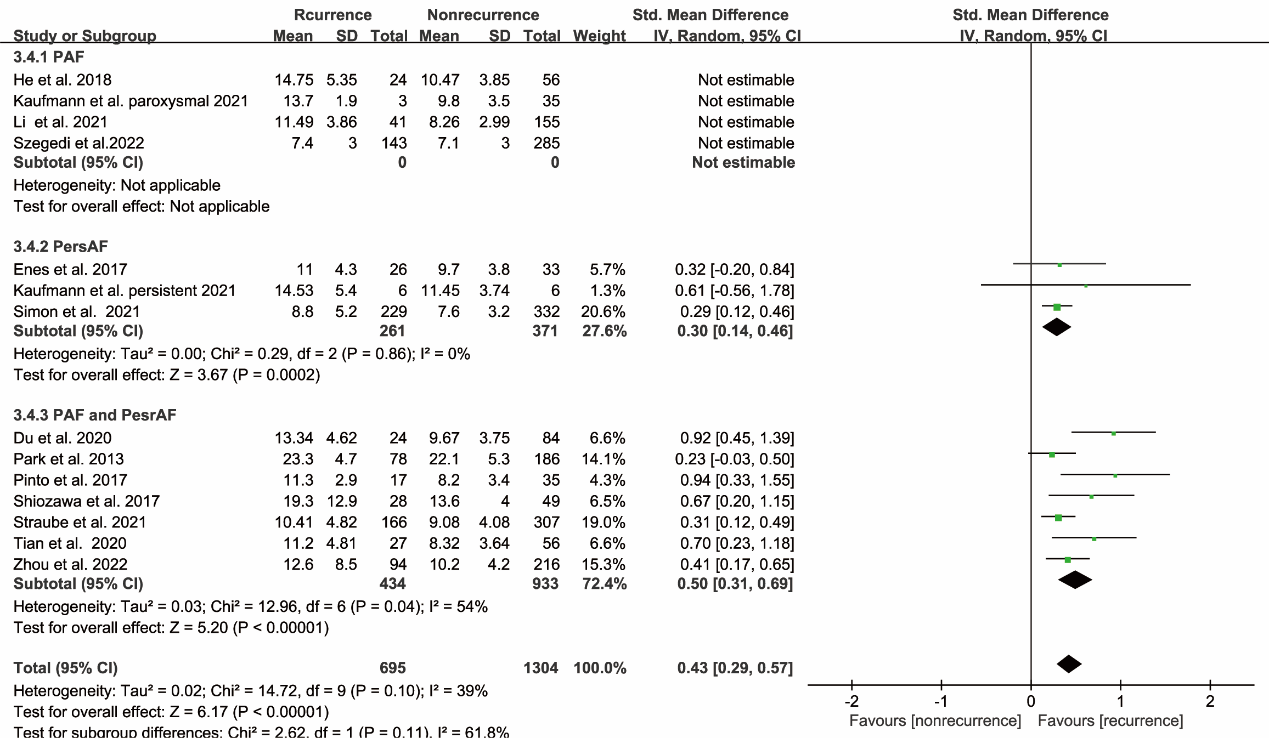


Supplementary Figure 2d


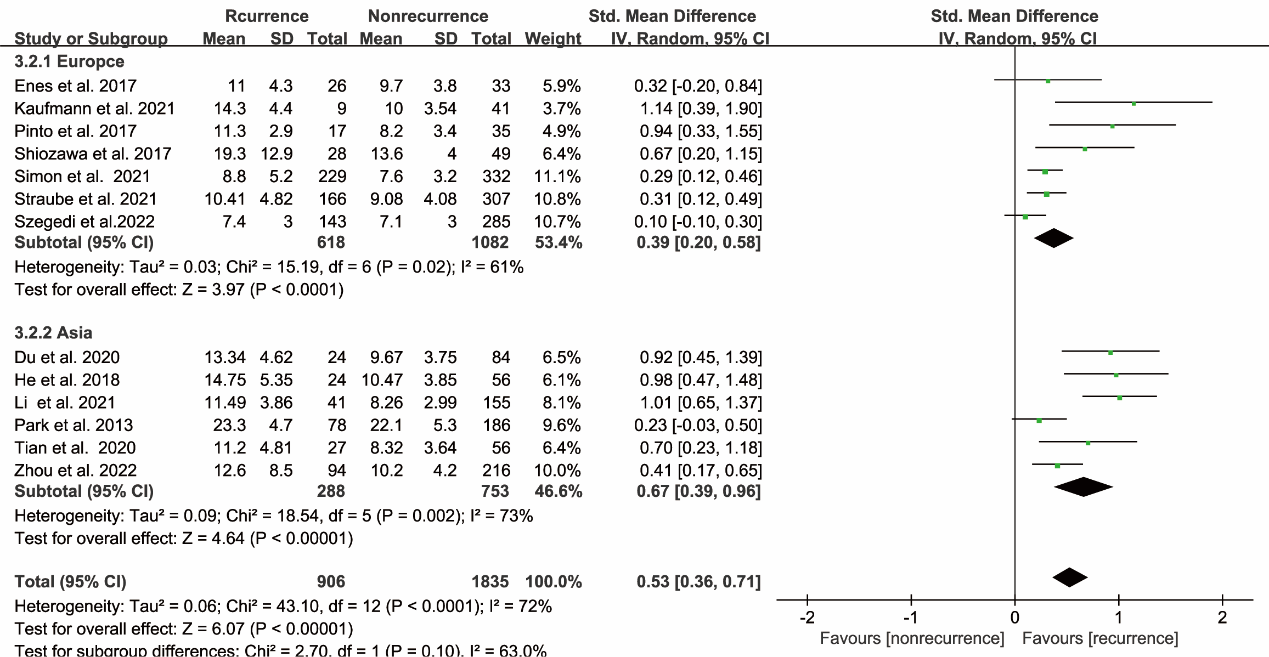


Supplementary Figure 2e


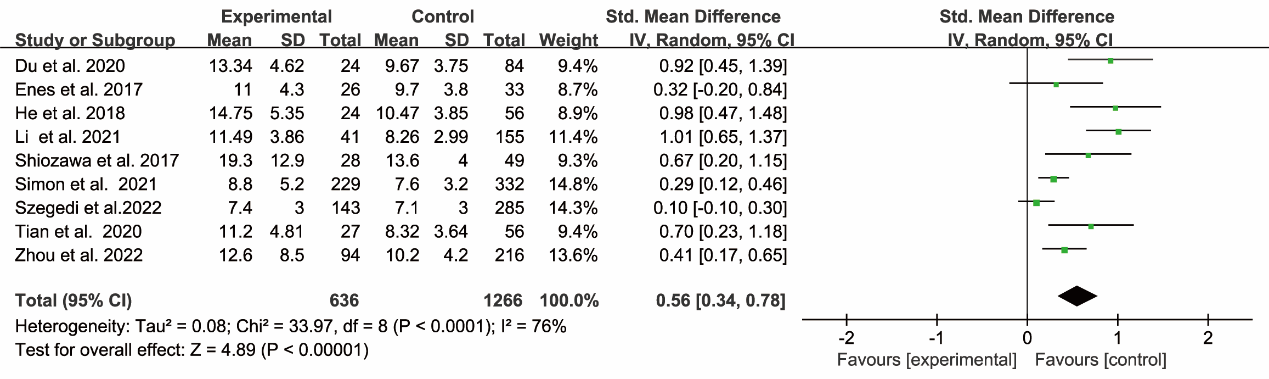


**Supplementary Figure 2** Subgroup analysis of the association of LAA volume with the post-ablation AF recurrence by a sample size (＜100, ＞100); b follow-up duration (≤12 months, > 12 months); c participants type (PAF: paroxysmal patients, Pers-AF: persistent AF patients, PAF and Pers-AF: both paroxysmal patients and persistent/nonPAF patients); d geographic region (Europe, Asia); e: forest plot showing the LAA volume at the 12-month of follow-up LAA: left atrial appendage.

Supplementary Figure 3a


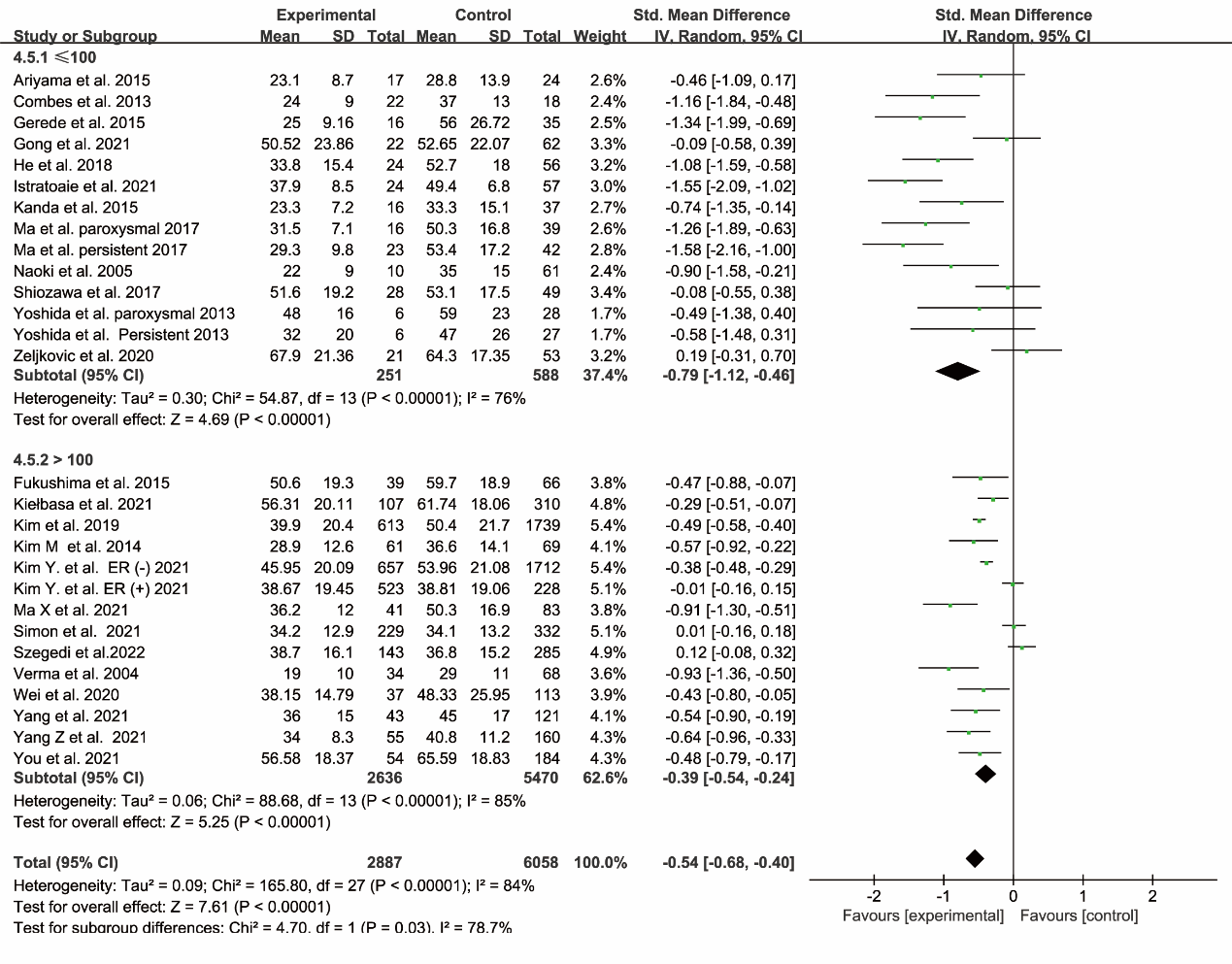


Supplementary Figure 3b


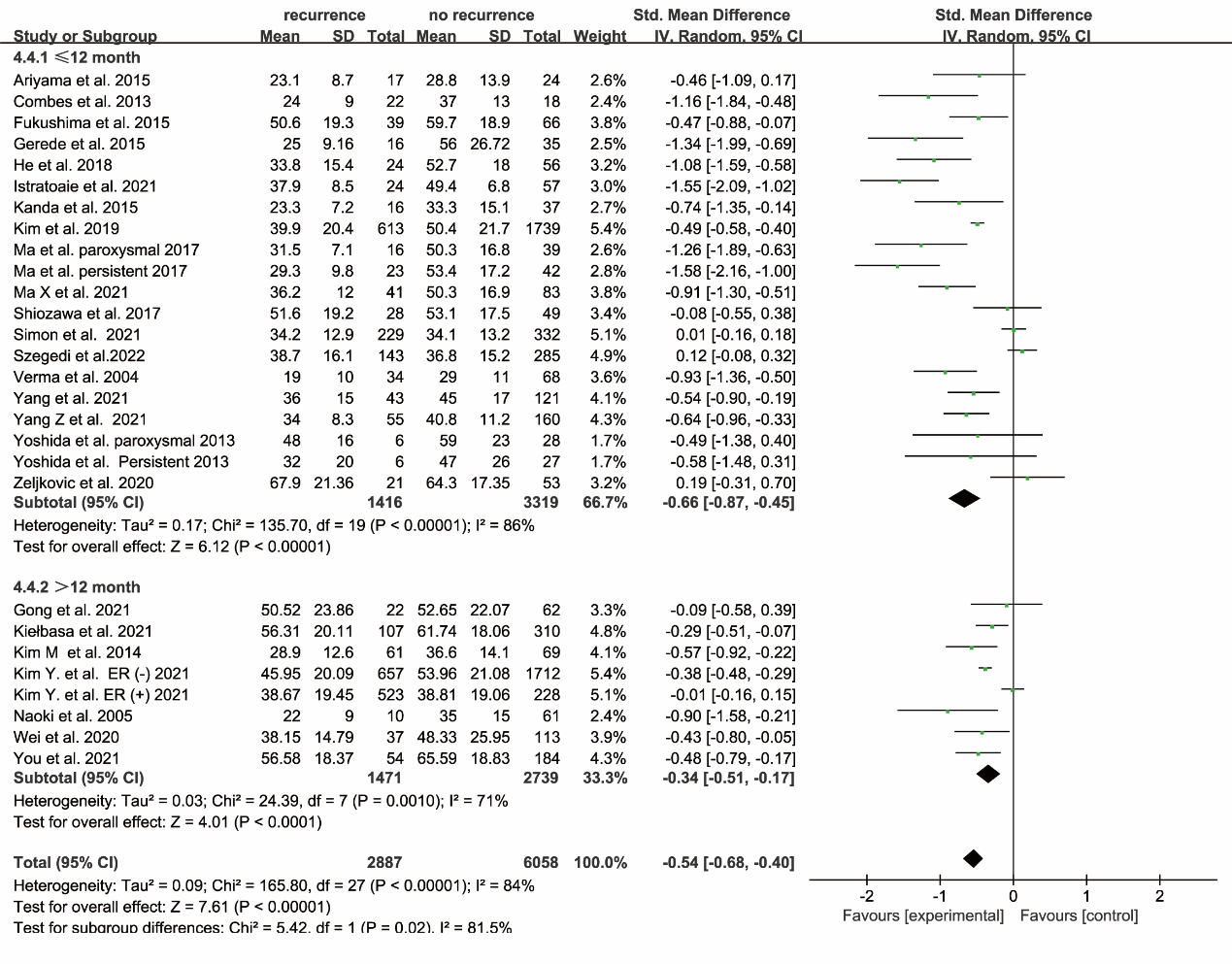


Supplementary Figure 3c

**
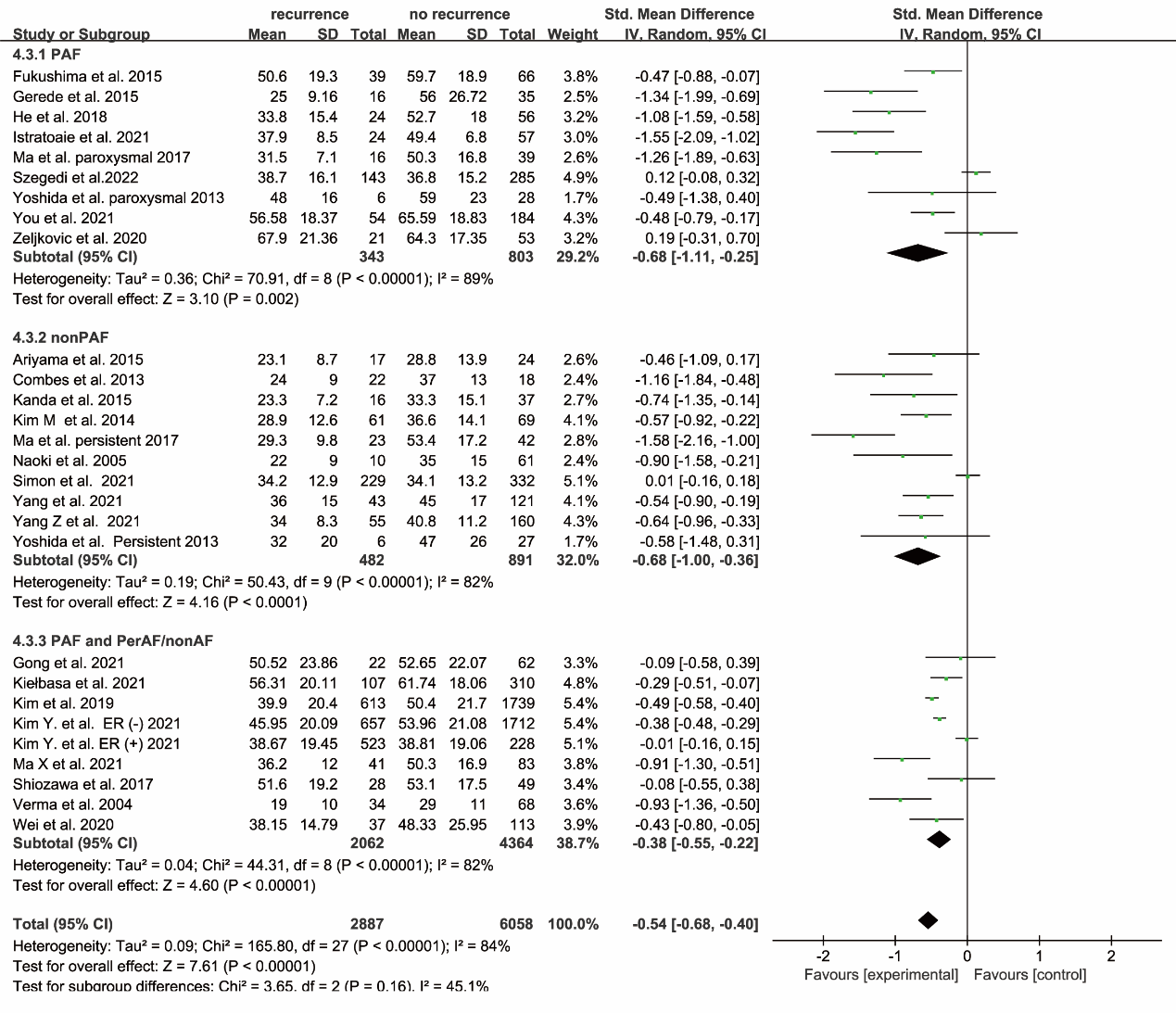
**

Supplementary Figure 3d

**
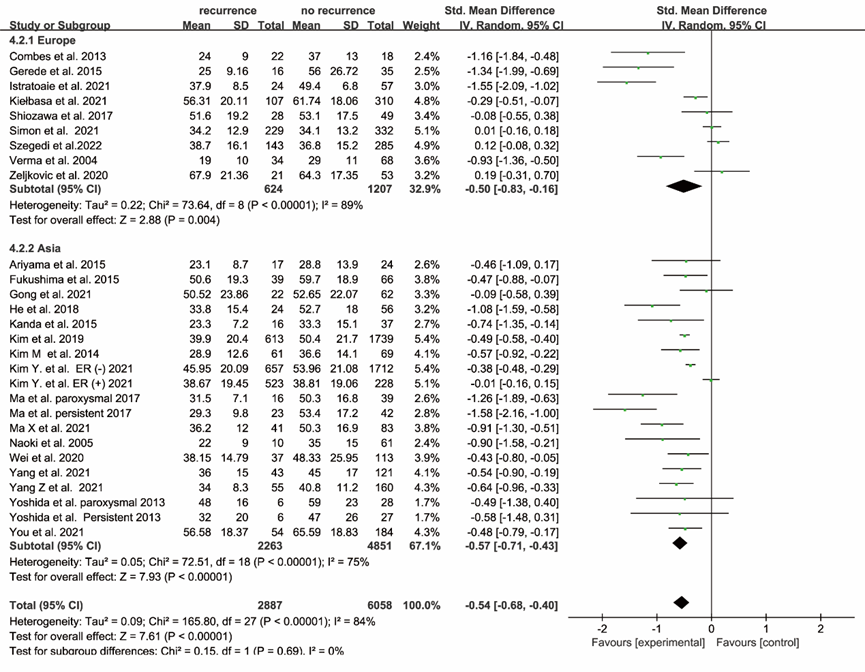
**

Supplementary Figure 3e

**
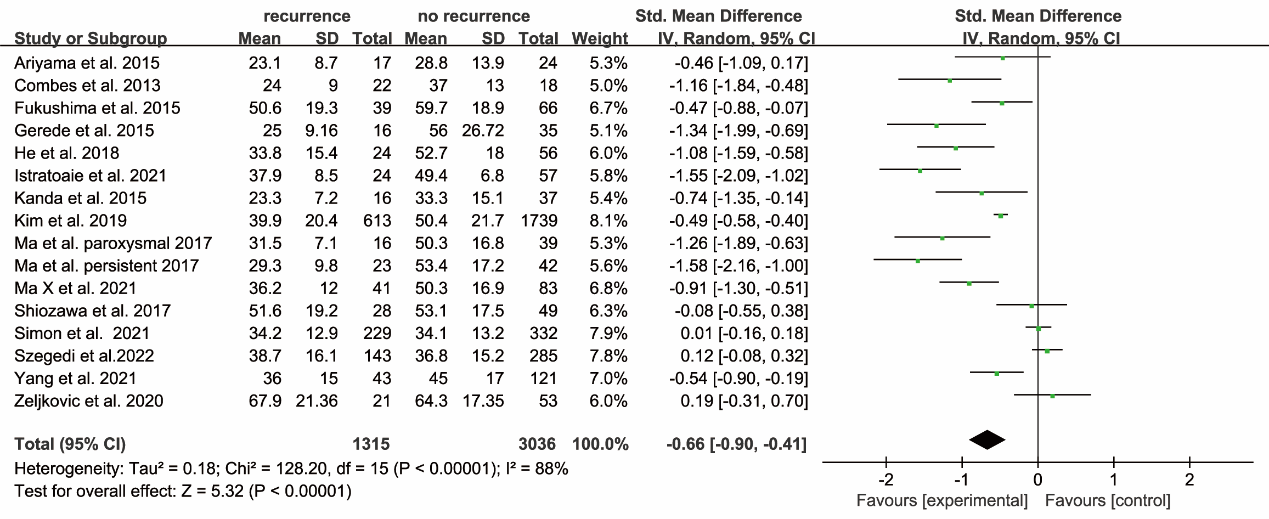
**

**Supplementary Figure 3** Subgroup analysis of the association of LAA emptying flow velocity with the post-ablation AF recurrence by a e sample size (＜100, ＞100); b follow-up duration (≤12 months, > 12 months); c participants type (PAF: paroxysmal patients, Pers-AF: persistent AF patients, PAF and Pers-AF: both paroxysmal patients and persistent/nonPAF patients); d geographic region (Europe, Asia); e: forest plot showing the LAA emptying flow velocity at the 12-month of follow-up LAA: left atrial appendage.

Supplementary Figure 4a


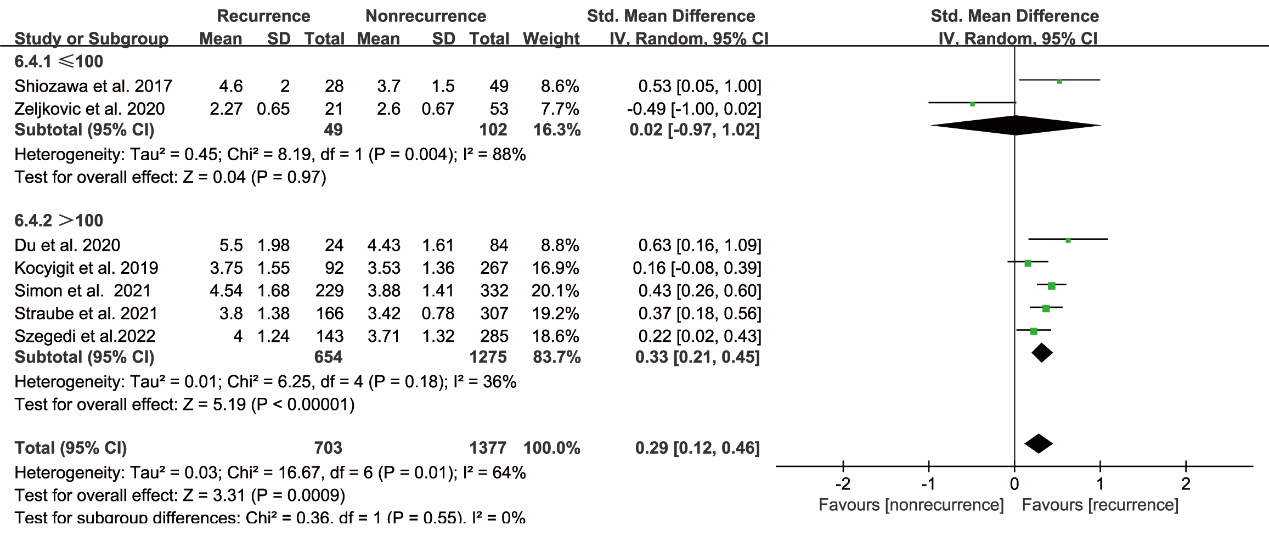


Supplementary Figure 4b


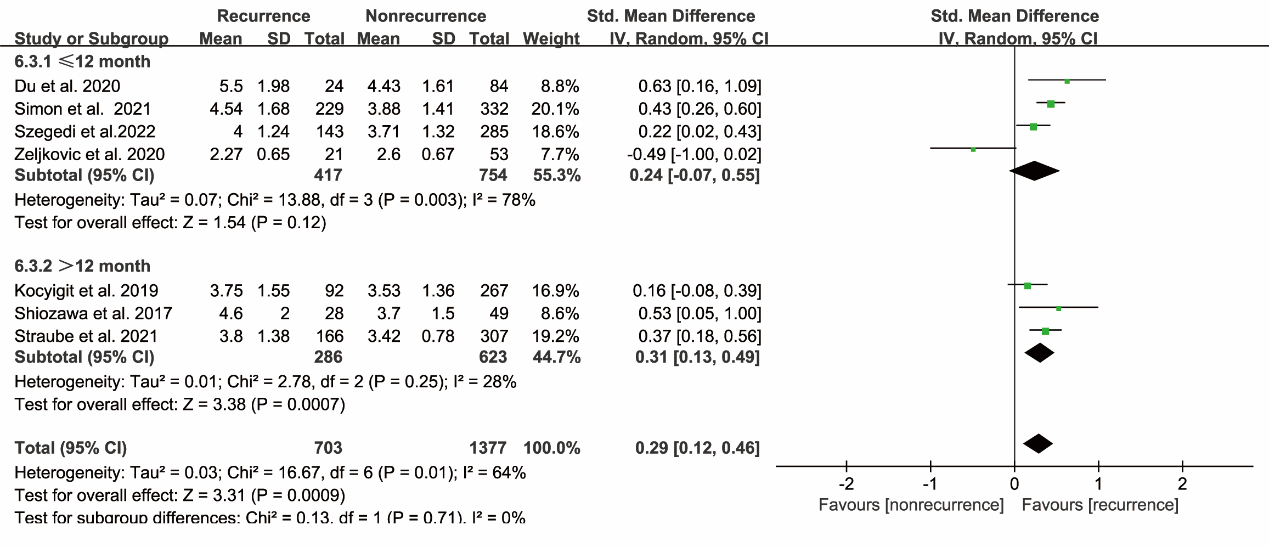


Supplementary Figure 4c


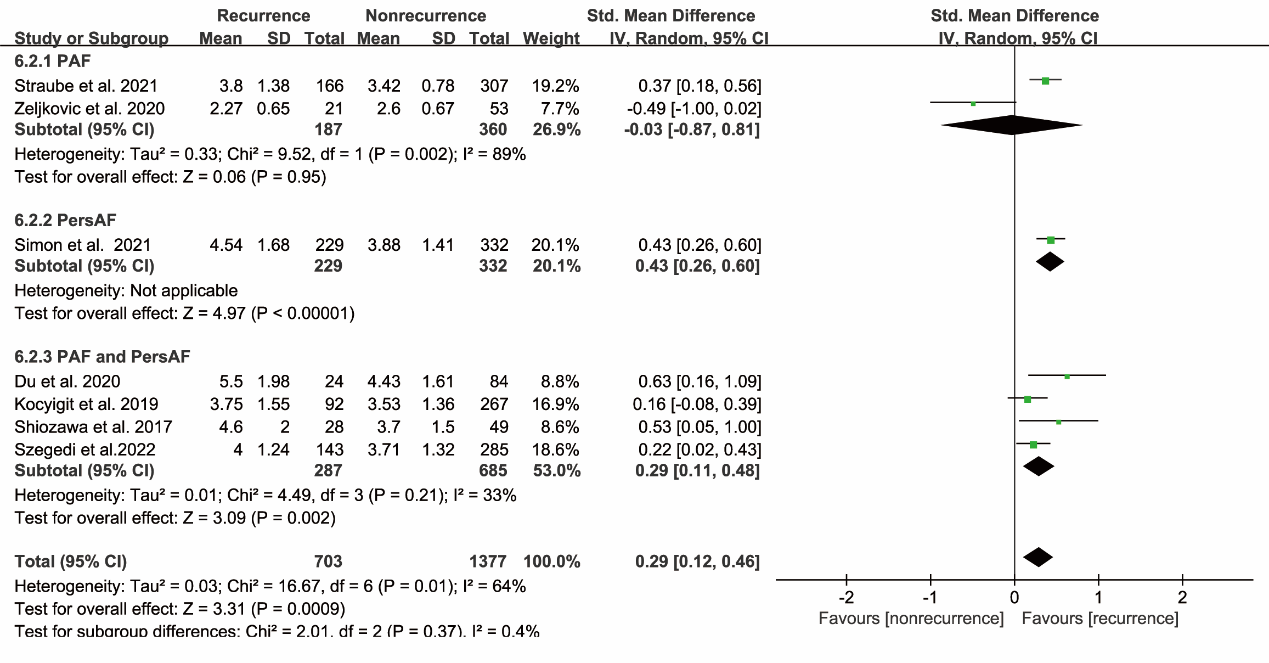


**Supplementary Figure 4** Subgroup analysis of the association of LAA-orifice area with the post-ablation AF recurrence by a sample size (＜100, ＞100); b follow-up duration (≤12 months, > 12 months); c participants AF type (PAF: paroxysmal patients, Pers-AF: persistent AF patients, PAF and Pers-AF: both paroxysmal patients and persistent/nonPAF patients); LAA: left atrial appendage.


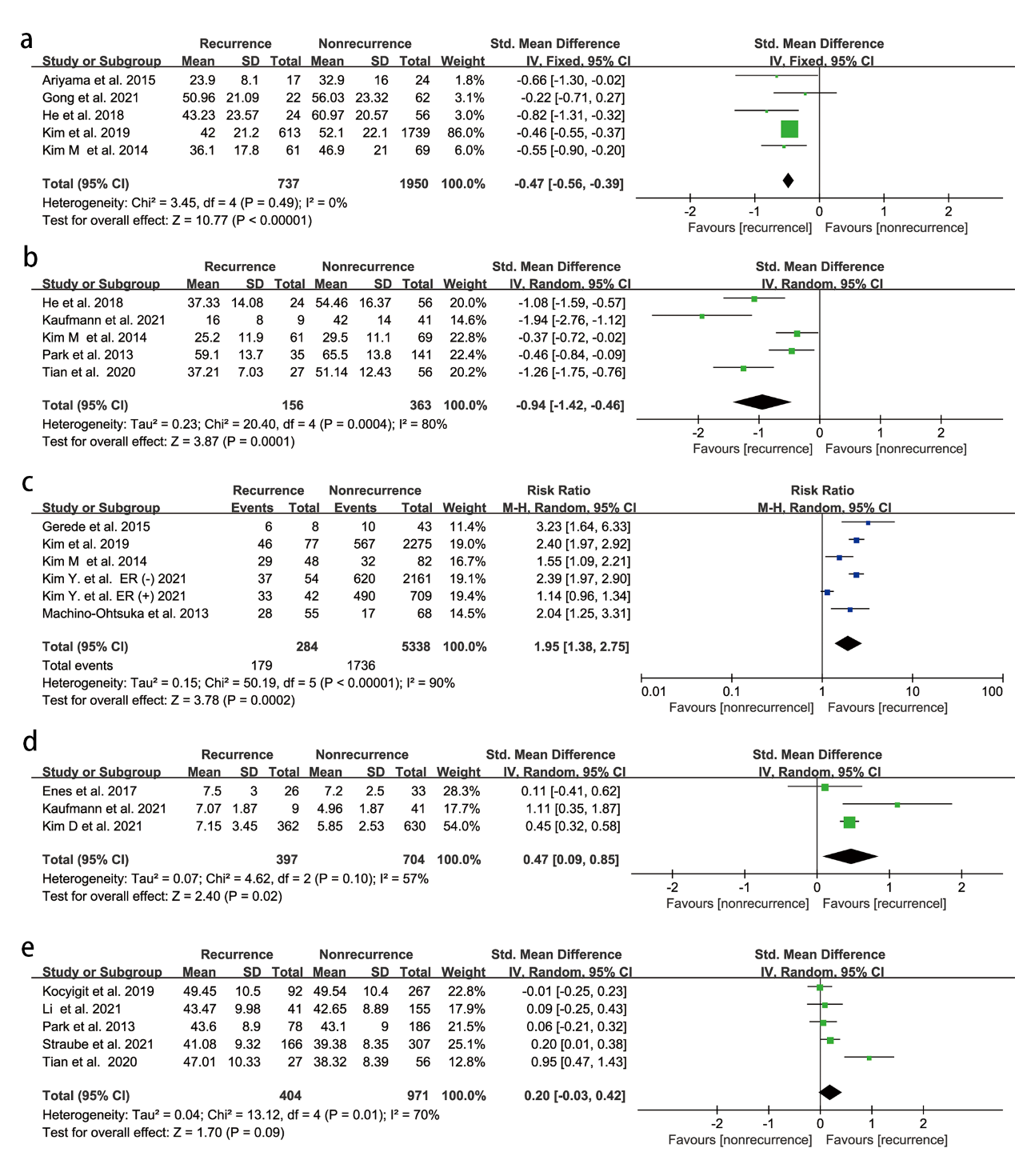


**Supplementary Figure 5** Overall effect analysis of the association of other LAA parameters with the post-ablation AF recurrence. a: LAA filling flow velocity; b: LAA ejection fraction;

c: left atrial spontaneous echo contrast; d: LAA volume index; e: LAA depth.

Supplementary Figure 6


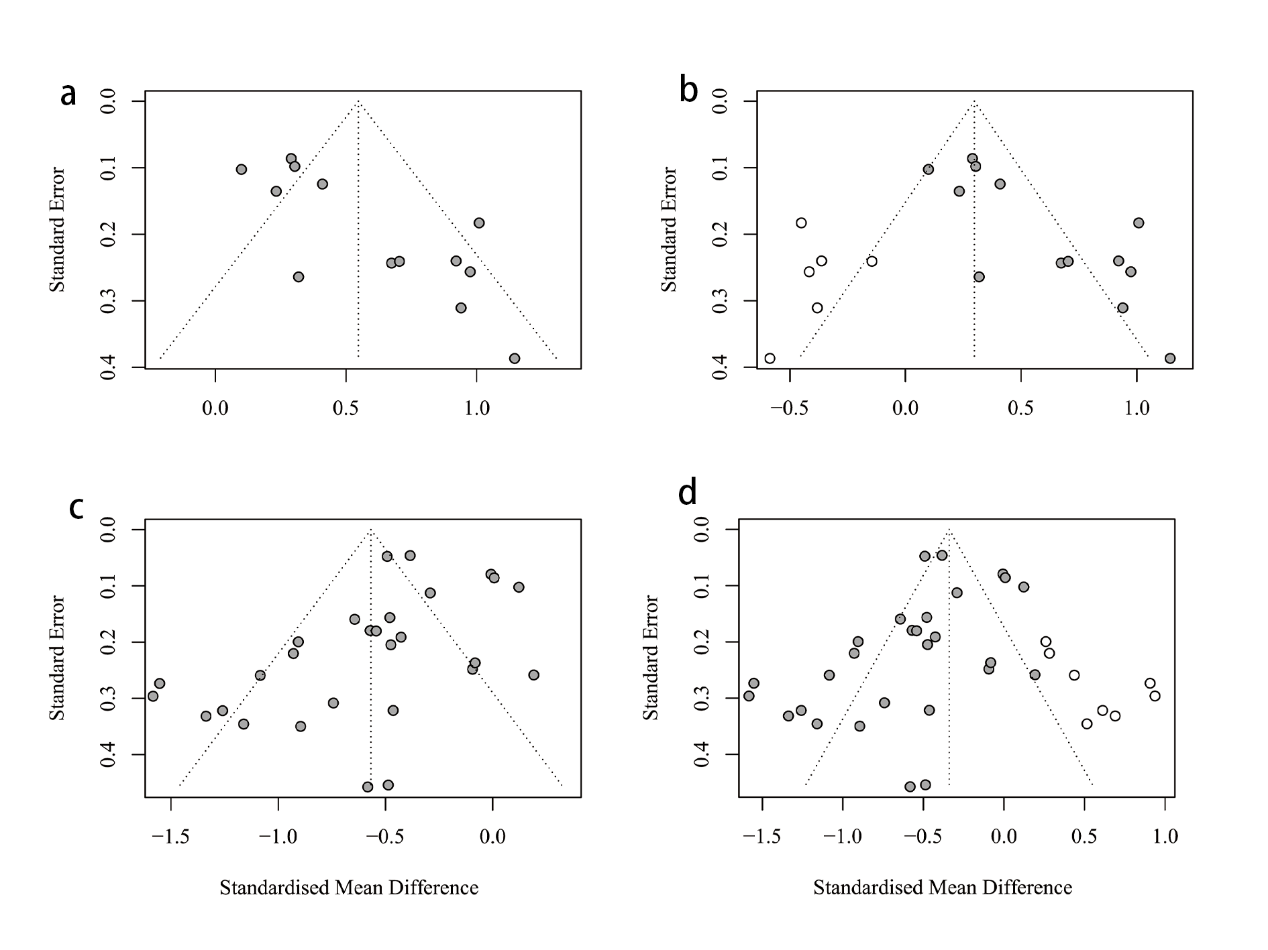


**Supplementary Figure 6** The Funnel plots of effect size against standard error and Egger’s test were used to explore the presence of publication bias. Each point represents a separate study for the indicated association. a. LAA volume and post-ablation AF recurrence (P= 0.0015); b. funnel plot with imputed number of studies (imputed studies and adjusted effect size are in white circle); c. LAA emptying flow velocity and post-ablation AF recurrence (P= 0.045); d. funnel plot with imputed number of studies (imputed studies and adjusted effect size are in white circle).

Supplementary Figure 7a





Supplementary Figure 7b


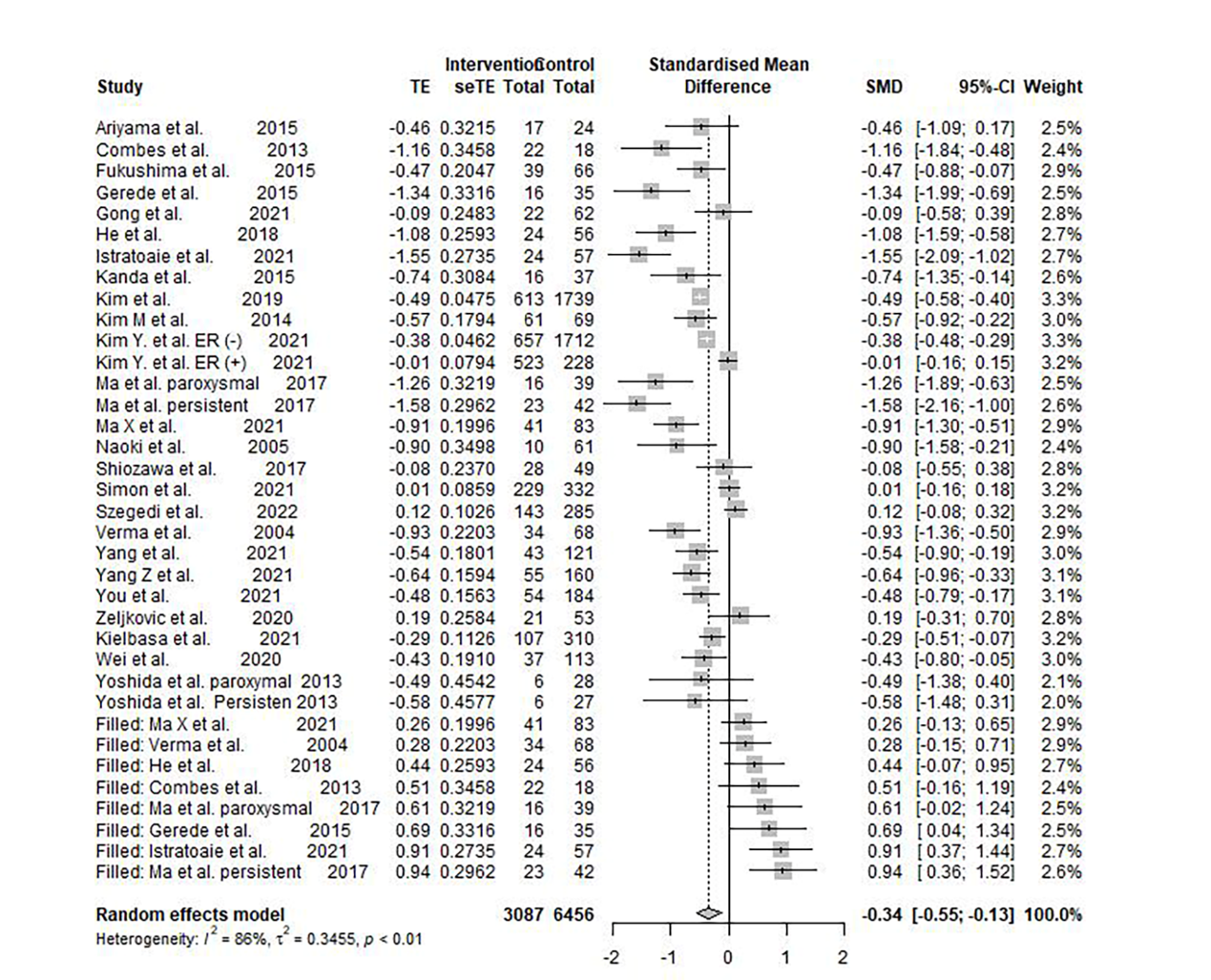


**Supplementary Figure 7** Forest plot showing the LAA volume(a) and emptying flow velocity(b) by the trim-and-fill method
